# A transgenic mouse model for studying HBV infection in neonate

**Published:** 2011-01-10

**Authors:** Zhuo Wang, Qiang Deng, Ke Lan

**Affiliations:** 1 Key Laboratory of Molecular Virology and Immunology, Institute Pasteur of Shanghai, Chinese Academy of Sciences, Shanghai 200025, PR China

## 

The human hepatitis B virus (HBV) infection is always a worldwide problem, especially the high risk infection of the neonate by chronically HBV infected mother. McGrane et al. [1] have demonstrated that the gene which is driven by the promoter of phosphoenolpyruvate carboxykinase (PEPCK) is mainly expressed in the mouse liver and immediately appears at parturition. We have constructed a transgenic mouse by using PEPCK promoter to drive the pre-S2 and S domain of HBV envelope protein to imitate HBV transmission from mother to child. We want to see whether hepatitis B surface antigen (HBsAg) can persistently exist or be cleared by immune system from the newborn mice.

## References

[B1] McGrane MM Yun JS Moorman AF Metabolic effects of developmental, tissue-, and cell-specific expression of a chimeric phosphoenolpyruvate carboxykinase (GTP)/bovine growth hormone gene in transgenic mice J Biol Chem 1990 265 22371 22379 1702419

